# Maternal Vitamin D Levels during Late Pregnancy and Risk of Allergic Diseases and Sensitization during the First Year of Life—A Birth Cohort Study

**DOI:** 10.3390/nu12082418

**Published:** 2020-08-12

**Authors:** Fui Chee Woon, Yit Siew Chin, Intan Hakimah Ismail, Amir Hamzah Abdul Latiff, Marijka Batterham, Yoke Mun Chan, on behalf of the MICOS Research Group

**Affiliations:** 1 Department of Nutrition and Dietetics, Faculty of Medicine and Health Sciences, Universiti Putra Malaysia, Selangor 43400, Malaysia; fuichee88@gmail.com (F.C.W.); cym@upm.edu.my (Y.M.C.); 2 Faculty of Engineering and Information Sciences, School of Mathematics and Applied Statistics, University of Wollongong, Wollongong, NSW 2522, Australia; 3 Research Centre of Excellence, Nutrition and Non-communicable Diseases, Faculty of Medicine and Health Sciences, Universiti Putra Malaysia, Selangor 43400, Malaysia; 4 Department of Paediatrics, Faculty of Medicine and Health Sciences, Universiti Putra Malaysia, Selangor 43400, Malaysia; intanhakimah@upm.edu.my; 5 Allergy & Immunology Centre, Pantai Hospital Kuala Lumpur, Kuala Lumpur 59100, Malaysia; allergymalaysia@gmail.com; 6 National Institute for Applied Statistics Research Australia, University of Wollongong, Wollongong, NSW 2522, Australia; marijka@uow.edu.au

**Keywords:** 25-hydroxyVitamin D, pregnancy, allergic diseases, sensitization, infant

## Abstract

Allergic diseases are the most common chronic illness in childhood. Findings from developed countries have reported associations between Vitamin D levels during pregnancy and offspring allergy risk. This prospective cohort study aimed to determine the associations between maternal Vitamin D levels during late pregnancy and allergic diseases in Malaysian infants during the first year of life. Serum 25(OH)D concentrations of 380 pregnant women in the third trimester were measured using a chemiluminescent immunoassay. Children’s allergic outcomes were assessed at 3, 6, and 12 months based on parental reports. Specific IgE antibodies against food and inhalant allergens were measured in infants at 12 months of age. A total of 43.2% pregnant women were Vitamin D deficient (<30 nmol/L) and 56.8% were nondeficient (≥30 nmol/L). A total of 27.6% of the infants had eczema, 6.1% had wheeze, 27.4% had food sensitization, 10.8% had inhalant allergen sensitization, and 3.8% had IgE-mediated food allergy during the first year of life. Compared with the nondeficient group, maternal Vitamin D deficiency in late pregnancy was not associated with any allergic outcomes after adjustment for potential confounding factors. In conclusion, the present study does not support an association between maternal Vitamin D levels in late pregnancy and allergic outcomes during the first year of life.

## 1. Introduction

Allergic diseases are the most common chronic illnesses in childhood and about 60% of allergies appear during first year of life [1]. Eczema and food allergy usually co-exist in early life, and eczema was proposed as an “entry point” for subsequent allergic diseases such as asthma and allergic rhinitis [1]. The global prevalence of allergic diseases has increased dramatically in recent decades and have affected about 20% of the world’s population [2]. The prevalence of allergic diseases including eczema, wheeze, and asthma, which were previously on the rise, have reached a plateau, or even started to decrease in some developed countries [3,4,5]. Conversely, emerging evidence shows that allergic disease prevalence, which was previously low, in developing countries continues to rise [6]. Development of eczema and food allergy early in life tends to increase the likelihood of developing other atopic diseases including asthma and allergic rhinitis in later childhood [1]. Apart from impaired quality of life, allergic diseases also place a profound social and financial burden on patients, their families, and society [2]. It is therefore important to identify the potentially modifiable risk factors of allergic diseases, so that early preventive measures can be taken.

The development of allergic diseases can be explained through the complex interplay between genetic inheritance and environmental exposures [2]. Although part of the increasing prevalence of allergic diseases in childhood can be explained by genetic predisposition, increased attention has been focused on the role of early life nutrition during the first 1000 days of life [7]. As diet is a modifiable risk factor, targeting the role of early life nutrition in the development of allergic diseases in children is essential for identifying potential primary prevention strategies. Vitamin D deficiency is one of the common micronutrient deficiencies during pregnancy [8]. Vitamin D, which has long been recognized for its importance in musculoskeletal health, has gained increased attention in recent years for its role in nonskeletal outcomes such as allergic diseases [9]. Findings from several birth cohorts suggested that maternal Vitamin D levels might play a role in the development of childhood allergic diseases [10,11,12]. During pregnancy, the fetus is totally dependent on the mother for an adequate supply of Vitamin D. Vitamin D in the fetus acquired from its mother through the placenta can affect immune development and subsequent risk of childhood allergy [13]. While findings from two birth cohorts revealed that a high maternal Vitamin D level is protective against childhood eczema, food allergy, and wheezing [10,12], others have shown that it is a risk factor for eczema and food allergy [11,14] or found no association [15]. Contradictory to the findings found in cohort studies, recent randomized controlled trials (RCTs) of Vitamin D supplementation in pregnancy have not proven to be effective against childhood allergies including eczema, food sensitization, wheeze, and asthma [16,17,18,19,20]. Results from previous studies have been controversial and most of these studies were conducted in developed countries. Therefore, more studies are needed to determine the role of Vitamin D levels during pregnancy in allergic diseases, especially in developing countries.

Despite the abundance of sunlight in Malaysia, a tropical country located right next to the equator, a high prevalence of Vitamin D deficiency has been reported among Malaysian pregnant women [21]. Considering the potential associations between maternal Vitamin D levels and allergy risk in children as reported in previous studies, Malaysian infants born to mothers with low Vitamin D levels during pregnancy might be at risk of allergy development. In view of the scarcity of prevalence data for childhood allergic diseases in Malaysia and that currently no study has examined their associations with maternal Vitamin D levels in this country, this study aims to determine the associations between maternal Vitamin D levels and the development of allergic diseases in infants during the first year of life.

## 2. Materials and Methods

### 2.1. Study Design and Study Population

This is a prospective cohort study conducted among pregnant women in late pregnancy participating in the Mother and Infant Cohort Study (MICOS) [22]. The protocol of the study and sample size calculation was previously described [22]. Between November 2016 and January 2018, the original cohort of 557 pregnant women was recruited at government Maternal and Child Health (MCH) clinics located in the state of Selangor and the Federal Territory of Kuala Lumpur, Malaysia. Participating pregnant women and their children were then followed up prospectively at 3, 6, and 12 months postpartum. The inclusion criteria were ≥18 years of age, gestation age ≥ 28 weeks at time of recruitment, singleton pregnancy, and receiving antenatal care at the selected clinics. The exclusion criteria were multiple pregnancies, delivery before 37 weeks of gestation, maternal immune deficiency, and fetal congenital anomalies. The study was approved by the Ethics Committee for Research Involving Human Subjects, Universiti Putra Malaysia [FPSK(FR16)P006] and the Medical Research and Ethics Committee, Ministry of Health Malaysia (NMRR-16-1047-30685). Written informed consent was obtained from all respondents.

### 2.2. Serum 25-Hydroxyvitamin D [25(OH)D] Analysis

Maternal serum 25(OH)D concentrations were measured once in late pregnancy. As detailed previously [22], a venous blood sample (2 mL) was collected from pregnant women during their routine antenatal check-up by trained nursing staff at the health clinics. When their blood was collected, the gestational age of the pregnant women was recorded. The blood sample was transferred to the blood collection tube and stored in the container provided by the laboratory at 2–8 °C. Blood samples were then sent to the laboratory (Pantai Premier Pathology Sdn. Bhd., Kuala Lumpur, Malaysia) within 24 h for further analysis. At the laboratory, the blood samples were analyzed by the trained laboratory staff using the Siemens ADVIA Centaur Vitamin D Total assay (Siemens, Tarrytown, NY, USA) to determine the serum 25(OH)D concentration. This assay has been standardized to the University of Ghent ID-LC/MS/MS reference measurement procedure and certified by the CDC Vitamin D Standardization Certification program [23]. Maternal serum 25(OH)D levels were categorized as deficient (<30 nmol/L) and nondeficient (≥30 nmol/L) [24]. Results of the serum 25(OH)D analysis were given to the mothers during the first postnatal follow-up at 3 months postpartum.

### 2.3. Allergic Sensitization

A peripheral venous blood sample of 1–2 mL was withdrawn via venepuncture in the dorsum of an infant’s hand by a trained medical assistant at the health clinic at 12 months follow-up. The blood sample was transferred into a serum separator tube and stored in the container provided by the laboratory at 2–8 °C. Blood samples were sent to the laboratory (Acutest Systems (M) Sdn. Bhd., Kuala Lumpur, Malaysia) within 24 h from the time of specimen collection for processing. The allergen-specific immunoglobulin E (IgE) levels against a panel of 19 types of food allergens (egg yolk, egg white, soybean, peanut, milk, clam, crab, shrimp, codfish, tuna, salmon, wheat, chicken, beef, rice, banana, orange, sesame seed, chocolate) and 16 types of inhalant allergens (house dust, *Dermatophagoides farinae*, *Dermatophagoides pteronyssinus*, *Blomia tropicalis*, Timothy grass, Bermuda grass, mucor, *Alternaria*, *Aspergillus*, *Candida*, *Cladosporium*, *Penicillium*, dog dander, cat dander, cockroach mix, and latex) were analyzed by the trained laboratory staff using the OPTIGEN Allergen Specific IgE Assay (Hitachi Chemical Diagnostics, Inc., Mountain View, CA). A level of specific IgE < 27 LU was rated as class 0, 27–65 LU as class 1, 66–142 LU as class 2, 143–242 LU as class 3, and >242 LU as class 4, respectively. Infants with a specific IgE level of class ≥ 1 were defined as having sensitization [25].

### 2.4. Allergic Outcomes

Allergic outcomes including eczema, IgE-mediated food allergy, and wheezing in infants were assessed at age 3, 6, and 12 months by trained researchers through face-to-face interviews with the mothers. Eczema was defined according to the UK Working Party’s Diagnostic Criteria for Atopic Dermatitis, namely having an itchy skin condition and fulfilling two or more of the following criteria: (i) family history of allergic disease; (ii) history dry skin; (iii) history of involvement of skin creases; and (iv) visible flexural eczema [26]. Infants with parent-reported food allergy symptoms who had a specific IgE level of class ≥ 1 to a specific food allergen were defined as having IgE-mediated food allergy. Food allergy symptoms were based on convincing clinical history that encompassed three of the following criteria: (i) parent reporting at least one recognized allergic symptom, which included localized symptoms (such as itching, sting/burning of the lips/mouth/throat, urticaria/hives, angioedema), abdominal symptoms (such as nausea, vomiting, crampy/colicky abdominal pain, diarrhea), respiratory symptoms (such as wheeze, stridor, watery rhinitis, redness of eyes/nose), skin symptoms (such as urticaria, itching, flushed skin, worsening eczema), or systemic reactions (such as anaphylaxis, syncope); (ii) parent reporting a temporal relationship of a reaction, with symptoms occurring within 2 h of food ingestion; and (iii) symptoms repeated each time the same food was consumed [2]. Wheeze was defined as the parental report of infants who had wheezing or whistling in the chest during the first year of life using the International Study of Asthma and Allergies in Childhood (ISAAC) questionnaire [27].

### 2.5. Covariates

Information on potential confounders associated with allergic outcomes [28] was collected. Information on maternal age, work status, ethnicity, educational level, maternal gestational age at blood withdrawal, parity, gestational age at delivery, birth weight, mode of delivery, and infant sex were obtained from clinic records. Meanwhile, information on monthly household income, maternal use of antibiotics during pregnancy, number of siblings, pets at home during the first year, infants’ daycare attendance during the first year, infant antibiotic use during the first year, exclusive breastfeeding, and family history of allergic disease were obtained via face-to-face interviews with the mothers. Family history of allergic disease was defined as any of the infant’s first-degree relatives having one or more histories of eczema, food allergy, asthma or allergic rhinitis.

### 2.6. Statistical Analysis

We used a log-binomial generalized linear mixed model (GLMM) to determine the associations between maternal Vitamin D levels and allergic diseases. Analysis was performed for the 380 mother–child pairs with complete data for three follow-ups. Pregnant women with deficient Vitamin D levels were considered as the “exposed” group, while those with nondeficient Vitamin D levels were considered as the “unexposed” group. Study sites and respondents were entered as random effects. Multivariable models were adjusted for potential confounding variables significantly associated with maternal Vitamin D levels and allergic outcomes (*p* < 0.05) identified from univariable models: ethnicity, gestational age at birth, mode of delivery, and antibiotic use in infants during the first year of life. We also performed multivariable models by adjusting additional confounding factors based on conceptual justification as suggested in previous literature [28]: maternal age, ethnicity, educational level, household income, work status, parity, antibiotic use during pregnancy, family history of allergic disease, gestational age at birth, infant birth weight, mode of delivery, sex, number of siblings, pet keeping, daycare attendance, antibiotic use in infants during the first year, and exclusive breastfeeding ≥ 6 months. All models were adjusted for gestational age at blood withdrawal and eczema status. Risk ratios (RRs) with a 95% confidence interval (CI) were calculated as the measure of associations between maternal Vitamin D levels and allergic diseases. Statistical analyses were performed using IBM SPSS Statistics 22 software (SPSS Inc., Chicago, IL, USA).

## 3. Results

### 3.1. Characteristics of the Mother–Child Pairs

**Figure 1:**
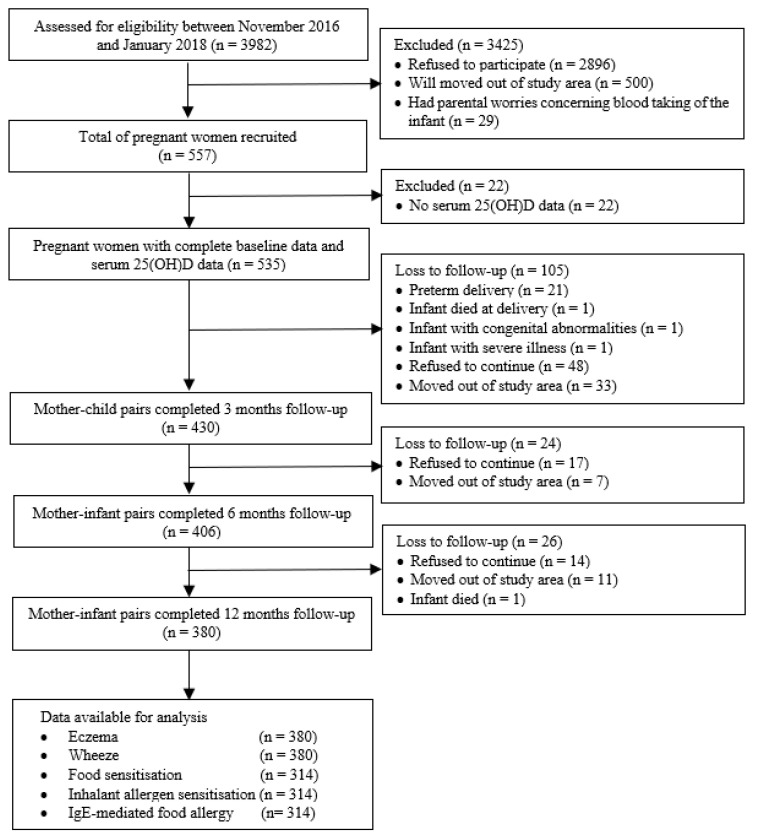
Flow chart of study respondents.

Of the 535 pregnant women who consented and completed baseline data at the third trimester, 430 mother–child pairs completed the 3 month follow-up, 406 completed the 6 month follow-up, and 380 completed the 12 month follow-up (Figure 1). The reasons for dropout include respondents who moved out of the study area and were unable to be contacted (51 mother–child pairs), those unwilling to continue their participation in the study or had parental worries concerning blood taking of their child (79 mothers), preterm delivery (21 infants), infant death (2 infants), or having been diagnosed with acute illness (2 infants).

Table 1 presents the characteristics of the study respondents. Of the 380 pregnant women, 43.2% were Vitamin D deficient, while 56.8% were nondeficient. Overall, the final cohort of the present study is representative of the original cohort as there were no significant differences in the characteristics of the respondents in terms of maternal age, ethnicity, educational level, work status, parity, family history of allergic disease, and maternal Vitamin D status during late pregnancy between the mother–child pairs who completed the 12 month follow-up (n = 380) and those loss to follow-up (n = 155) except for with monthly household income.

### 3.2. Allergic Outcomes in Infants

Of the 380 infants, 27.6% developed eczema and 6.1% developed wheeze, respectively, during the first year of life (Table 2). Of the 314 infants who undertook the allergen-specific IgE test at 12 months of age, 27.4% were sensitized to at least one of the food allergens tested and 10.8% were sensitized to at least one of the inhalant allergens tested. The top three food allergens sensitized by infants at 12 months of age were beef (14.3%), peanut (10.8%), and egg white (7.0%), while the top three inhalant allergens were *Dermatophagoides farinae* (6.4%), *Dermatophagoides pteronyssinus* (5.4%), and *Blomia tropicalis* (4.1%). The prevalence of IgE-mediated food allergy was 3.8%, with 3.2% egg allergy, 1.0% cow’s milk allergy, 0.6% wheat allergy, and 0.3% soy allergy.

### 3.3. Associations between Maternal Vitamin D Levels and Allergic Diseases

Table 3 shows the associations of maternal Vitamin D levels with each of the allergic outcomes. We observed no associations of maternal Vitamin D deficient in late pregnancy with any of the allergic outcomes in infants during the first year of life, compared with the nondeficient group. These null associations remained after adjustment for potential confounding factors.

## 4. Discussion

The results of this prospective cohort study suggest that maternal Vitamin D levels in late pregnancy were not associated with offspring eczema, wheeze, food sensitization, inhalant allergen sensitization, and IgE-mediated food allergy during the first year of life when adjusted for a range of potential confounding factors.

The prevalence of infantile eczema (27.6%), wheezing (6.1%), and inhalant allergen sensitization (10.8%) in the present study is comparable with the parent-reported eczema (20.9%), wheezing (9.8%), and aeroallergen sensitization (11.2%) in infants at 18 months of age in the Singapore GUSTO cohort study [29,30]. Similarly, the IgE-mediated food allergy prevalence (3.8%) in the present study is in line with the prevalence of IgE-mediated food allergy (2.9%) in Singaporean infants aged 12 months [31]. Our study suggests that Malaysian children are at high risk of developing allergies in early life, and these health issues should be given special attention by health professionals.

Studies that assessed the associations between maternal Vitamin D levels and childhood food allergy and sensitization reported inconsistent results [10,11,15,32]. The Taiwan PATCH cohort study found that high maternal Vitamin D levels (≥75 nmol/L) were protective against food sensitization in children at age 1.5 and 2 years [10]. In contrast, the German LINA cohort study revealed that higher maternal Vitamin D levels were associated with an increased risk of food allergy and food sensitization in children at the age of 2 years [11]. In line with our findings, the Cork BASELINE birth cohort and the GUSTO study found that maternal Vitamin D levels were not associated with childhood food allergy food sensitization [15,32]. Similar findings were reported in an RCT conducted in the UK that failed to detect an effect of prenatal Vitamin D supplementation on the risk of food allergy in infants at 3 years of age [16]. It should be noted that the comparison of findings across studies might be difficult due to differences in terms of length of follow-up, the period of pregnancy at which maternal Vitamin D levels were measured, methods of food allergy and food sensitization measurement such as physician-diagnosed food allergy [11,15], skin prick test [15,32], or specific IgE-confirmed food sensitization [10]. In the present study, we did not observe an association between maternal Vitamin D levels and childhood food allergy, and we therefore speculate that other factors such as genetic factors may play a more important role in explaining this association. The Boston birth cohort conducted by Liu et al. [33] showed that cord blood Vitamin D levels were not associated with food sensitization in early childhood; however, a significant inverse association was found in children with particular genotypes. Therefore, further studies are needed to explore the interactions between genetic factors and Vitamin D levels in explaining their relationships with childhood food allergy.

There is lack of consistent findings addressing the associations between maternal Vitamin D levels and childhood eczema [14,15,32,34]. The inconsistent findings may be explained by the “U-shape” associations, suggesting that both lower and higher levels of Vitamin D are associated with a higher risk of eczema [14,34]. The UK birth cohort found that maternal 25(OH)D concentrations > 75 nmol/L were associated with increased offspring eczema risks at 9 months of age [14]. Another study reported that mid-pregnancy 25(OH)D concentrations < 25 nmol/L were associated with the increased risk of eczema in children ≤ 3 years of age [34]. In contrast, we found no associations between maternal Vitamin D levels and the development of eczema in childhood, which is in line with the findings reported in the Cork BASELINE birth cohort [15] and the Generation R study [35]. Similarly, results from several RCTs have not found a protective role of maternal Vitamin D supplementation during pregnancy in the risk of eczema in infants and children [16,17,18,19]. Apart from methodological differences across studies, the null associations between maternal Vitamin D levels and eczema in the present study may be explained by genetic factors, which play a more important role in the development of childhood eczema. Evidence showed that mutations in the filaggrin gene have been strongly associated with the development of eczema [36,37]. Therefore, the potential for Vitamin D to interact with genetic factors in explaining the development of childhood eczema should be considered in future studies.

Consistent with previous studies [20,38,39], we found no associations between maternal Vitamin D levels in late pregnancy and wheeze and inhalant allergen sensitization in infants during the first year of life. Similarly, findings from several RCTs showed that maternal Vitamin D supplementation during pregnancy did not pose an effect on the development of wheeze and inhalant allergen sensitization in infants during the first 3 years of life [16,17,18,19,20]. In contrast to our findings, Rothers et al. [40] reported that both low (<50 nmol/L) and high (>100 nmol/L) levels of cord blood Vitamin D levels were associated with increased specific IgE levels with certain inhalant allergens in children at 5 years of age. Another study demonstrated that low maternal Vitamin D levels (<50 nmol/L) were associated with the higher risk of aeroallergen sensitization during the first 2 years of life [10]. As findings from the birth cohorts are inconsistent and previous RCTs failed to demonstrate the protective role of prenatal Vitamin D supplementation on wheeze and inhalant allergen sensitization in infants, further studies are needed to determine the combined effects of prenatal and postnatal Vitamin D status on the development of allergic diseases in offspring.

To the best of knowledge, the present study is the first prospective cohort study to report the relationship between maternal Vitamin D levels and infants’ allergic outcomes in Malaysia. The strengths of our study include a longitudinal study design which enables information on a large number of potential confounders to be recorded and adjusted in multivariable analyses. Parental reports of allergic outcomes, rather than direct assessment by physicians, are the major limitation of this study. Previous studies have demonstrated that both lower and higher maternal Vitamin D levels were associated with an increased risk of allergic diseases in offspring [14,34,40]. In the present study, we managed to determine the associations between low levels of maternal Vitamin D and infants’ allergic outcomes but were unable to assess the outcomes for high maternal Vitamin D levels due to low numbers of pregnant women with sufficient Vitamin D levels, which may lead to insufficient statistical power to detect significant associations with allergic outcomes. Similarly, the low number of infants with wheeze and IgE-mediated food allergy reported in the present study may lead to insufficient statistical power to detect their associations with maternal Vitamin D levels. Attrition is a concern in prospective cohort studies which may lead to selection bias. However, there were no significant differences in the majority of the characteristics between the respondents who completed the study and those loss to follow-up, suggesting limited bias. The present study was able to report the relationship between low Vitamin D levels in the third trimester pregnant mothers and allergic disease development in infants from Selangor and Kuala Lumpur—the two most urbanized states in Malaysia. However, the findings might not be generalizable to the other populations. In the present study, maternal Vitamin D levels were measured once in late pregnancy, as evidence has shown that maternal 25(OH)D levels were the highest in late pregnancy and were associated with infants’ serum 25(OH)D [41,42,43]. However, our study was unable to determine the changes in maternal Vitamin D levels over the course of pregnancy and their effects on the study outcomes.

## 5. Conclusions

In conclusion, our results suggest that maternal Vitamin D levels in late pregnancy are not associated with allergic outcomes in infants during the first year of life. Further studies are needed to explore the role of Vitamin D in childhood allergies in combination with other environmental and genetic factors.

## Figures and Tables

**Table 1:** Characteristics of the mother–child pairs.

	Total			Maternal 25(OH)D Levels		
Characteristics	Included in Age 12 Month Analysis (n = 380)	Loss to Follow-Up (n = 155)	*p*-Value	Deficient < 30 nmol/L (n = 164)	Nondeficient ≥ 30 nmol/L (n = 216)	*p*-Value
Maternal 25(OH)D levels						
Deficient (<30 nmol/L)	164 (43.2)	63 (40.6)	0.594			
Nondeficient (≥30 nmol/L)	216 (56.8)	92 (59.4)				
Gestational age at blood withdrawal (weeks)						
Median (IQR)	32 (29, 36)	31 (28–35)	0.013			
**Family characteristics**						
Maternal age (years)	30.1 ± 4.2	29.6 ± 4.0	0.225	30.0 ± 4.0	30.2 ± 4.3	0.591
Maternal ethnicity, Malay (%)	350 (92.1)	143 (92.3)	0.952	161 (98.2)	189 (87.5)	0.001
Maternal educational level, higher (%)	312 (82.1)	126 (81.3)	0.824	129 (78.7)	183 (84.7)	0.127
Monthly household income						
Low (< RM 2300)	52 (13.7)	40 (25.8)	0.003	26 (15.9)	26 (12.0)	0.062
Moderate (RM 2300–5599)	209 (55.0)	72 (46.5)		97 (59.1)	112 (51.9)	
High (≥RM 5600)	119 (31.3)	43 (27.7)		41 (25.0)	78 (36.1)	
Maternal work status, working (%)	267 (70.3)	103 (66.5)	0.387	118 (72.0)	149 (69.0)	0.530
Parity, multiparous (%)	226 (59.5)	83 (53.5)	0.208	101 (61.6)	125 (57.9)	0.465
Family history of allergic disease, yes (%)	257 (67.6)	98 (63.2)	0.328	109 (66.5)	148 (68.5)	0.672
Maternal antibiotics use during pregnancy, yes (%)	44 (11.6)	6 (12.0) ^a^	0.930	37 (22.6)	56 (25.9)	0.450
Pet keeping, yes (%)	93 (24.5)	^−^	−	13 (7.9)	31 (14.4)	0.053
**Infant characteristics**						
Gestational age at birth (weeks)	38.9 ± 1.1	38.8 ± 1.0 ^a^	0.579	38.8 ± 1.1	38.9 ± 1.2	0.867
Birth weight (kg)	3.1 ± 0.4	3.1 ± 0.4 ^a^	0.845	3.1 ± 0.4	3.1 ± 0.4	0.620
Mode of delivery, vaginal (%)	278 (73.2)	36 (72.0) ^a^	0.862	119 (72.6)	159 (73.6)	0.819
Sex, male (%)	190 (50.0)	31 (62.0) ^a^	0.110	77 (47.0)	113 (52.3)	0.300
Older siblings, yes (%)	226 (59.5)	83 (53.5)	0.208	101 (61.6)	125 (57.9)	0.465
Daycare attendance, yes (%)	207 (54.5)	−	−	82 (50.0)	125 (57.9)	0.127
Antibiotic use, yes (%)	224 (58.9)	−	−	93 (56.7)	131 (60.6)	0.439
Exclusive breastfeeding till 6 months (%)	177 (46.6)	13 (50.0) ^b^	0.735	74 (45.1)	103 (47.7)	0.620

Data shown are the mean ± standard deviation for the continuous variables and number (percentage) of respondents for categorical variables. *p*-values for difference were determined by a Chi-square test for categorical variables and an independent *t*-test for two independent samples. RM, Ringgit Malaysia (1 USD = RM 4.28, as of June 23, 2020). ^a^ Data available for 50 mother–child pairs who completed the 3 month follow-up. ^b^ Data available for 26 mother–child pairs who completed the 6 month follow-up.

**Table 2:** Allergic diseases in infants during the first year of life.

Allergic Diseases	N (%)
Eczema in the past 12 months (n = 380)	105 (27.6)
Wheeze in the past 12 months (n = 380)	23 (6.1)
Food sensitization at 12 months (n = 314) ^1^	86 (27.4)
Beef (n = 314)	45 (14.3)
Peanut (n = 314)	34 (10.8)
Egg white (n = 314)	22 (7.0)
Egg yolk (n = 314)	10 (3.2)
Soya (n = 314)	14 (4.5)
Cow’s milk (n = 314)	7 (2.2)
Shrimp (n = 314)	6 (1.9)
Crab (n = 314)	6 (1.9)
Clam (n = 314)	4 (1.3)
Codfish (n = 314)	4 (1.3)
Wheat (n = 314)	4 (1.3)
Salmon (n = 314)	3 (1.0)
Chocolate (n = 314)	2 (0.6)
Rice (n = 314)	2 (0.6)
Tuna (n = 314)	2 (0.6)
Chicken (n = 314)	1 (0.3)
Orange (n = 314)	1 (0.3)
Inhalant allergen sensitization at 12 months (n = 314) ^1^	34 (10.8)
*Dermatophagoides farinae* (n = 314)	20 (6.4)
*Dermatophagoides pteronyssinus* (n = 314)	17 (5.4)
*Blomia tropicalis* (n = 314)	13 (4.1)
*Candida* (n = 314)	7 (2.2)
Cat dander (n = 314)	7 (2.2)
House dust (n = 314)	6 (1.9)
Dog dander (n = 314)	4 (1.3)
Cockroach mix (n = 314)	4 (1.3)
*Penicillium* (n = 314)	3 (1.0)
*Cladosporium* (n = 314)	2 (0.6)
*Aspergillus* (n = 314)	1 (0.3)
Bermuda grass (n = 314)	1 (0.3)
IgE-mediated food allergy at 12 months (n = 314)	12 (3.8)
Eggs (n = 314)	10 (3.2)
Cow’s milk (n = 314)	3 (1.0)
Wheat (n = 314)	2 (0.6)
Soy (n = 314)	1 (0.3)

Data shown are the number (percentage) of respondents. ^1^ Allergens with 0% respondents were not shown.

**Table 3:** Associations between maternal 25(OH)D levels and allergic diseases in infants during the first year of life.

Allergic Outcomes	Crude		Adjusted ^1^		Adjusted ^2^	
	RR (95% CI)	*p*-Value	RR (95% CI)	*p*-Value	RR (95% CI)	*p*-Value
Eczema (n = 380)						
Nondeficient (≥30 nmol/L)	1		1		1	
Deficient (<30 nmol/L)	1.02 (0.77–1.35)	0.884	1.04 (0.79–1.38)	0.770	1.10 (0.83–1.46)	0.495
Wheeze (n = 380)						
Nondeficient (≥30 nmol/L)	1		1		1	
Deficient (<30 nmol/L)	1.01 (0.48–2.13)	0.973	1.04 (0.50–2.18)	0.915	1.10 (0.61–2.00)	0.755
Food allergen sensitization (n = 314)						
Nondeficient (≥30 nmol/L)	1		1		1	
Deficient (<30 nmol/L)	1.22 (0.85–1.75)	0.282	1.08 (0.76–1.54)	0.650	1.05 (0.75–1.48)	0.782
Inhalant allergen sensitization (n = 314)						
Nondeficient (≥30 nmol/L)	1		1		1	
Deficient (<30 nmol/L)	0.58 (0.29–1.16)	0.122	0.58 (0.29–1.15)	0.121	0.59 (0.29–1.19)	0.137
IgE-mediated food allergy (n = 314)						
Nondeficient (≥30 nmol/L)	1		1		1	
Deficient (<30 nmol/L)	0.54 (0.18–1.62)	0.269	0.64 (0.30–1.40)	0.268	0.68 (0.31–1.53)	0.355

CI, confidence interval; RR, relative risk. ^1^ Model was adjusted for ethnicity, gestational age at blood withdrawal, gestational age at birth, mode of delivery, and antibiotic use in infants during the first year. ^2^ Model was adjusted for maternal age, ethnicity, educational level, household income, work status, parity, antibiotic use during pregnancy, gestational age at blood withdrawal, family history of allergic disease, gestational age at birth, infant birth weight, mode of delivery, sex, number of siblings, pet keeping, daycare attendance, antibiotic use during the first year, and exclusive breastfeeding ≥ 6 months. Food allergy, wheeze, and allergen sensitization outcomes were adjusted for eczema status.
